# IoTDS: A One-Class Classification Approach to Detect Botnets in Internet of Things Devices

**DOI:** 10.3390/s19143188

**Published:** 2019-07-19

**Authors:** Vitor Hugo Bezerra, Victor Guilherme Turrisi da Costa, Sylvio Barbon Junior, Rodrigo Sanches Miani, Bruno Bogaz Zarpelão

**Affiliations:** 1 Computer Science Department, State University of Londrina (UEL), Londrina PR 86057-970, Brazil; 2 School of Computer Science, Federal University of Uberlândia (UFU), Uberlândia MG 38400-902, Brazil

**Keywords:** Internet of Things, botnet, anomaly detection, host-based

## Abstract

Internet of Things (IoT) devices have become increasingly widespread. Despite their potential of improving multiple application domains, these devices have poor security, which can be explored by attackers to build large-scale botnets. In this work, we propose a host-based approach to detect botnets in IoT devices, named IoTDS (Internet of Things Detection System). It relies on one-class classifiers, which model only the legitimate device behaviour for further detection of deviations, avoiding the manual labelling process. The proposed solution is underpinned by a novel agent-manager architecture based on HTTPS, which prevents the IoT device from being overloaded by the training activities. To analyse the device’s behaviour, the approach extracts features from the device’s CPU utilisation and temperature, memory consumption, and number of running tasks, meaning that it does not make use of network traffic data. To test our approach, we used an experimental IoT setup containing a device compromised by bot malware. Multiple scenarios were made, including three different IoT device profiles and seven botnets. Four one-class algorithms (Elliptic Envelope, Isolation Forest, Local Outlier Factor, and One-class Support Vector Machine) were evaluated. The results show the proposed system has a good predictive performance for different botnets, achieving a mean F1-score of 94% for the best performing algorithm, the Local Outlier Factor. The system also presented a low impact on the device’s energy consumption, and CPU and memory utilisation.

## 1. Introduction

Internet of Things (IoT) has evolved very quickly since this term was coined by Ashton in 1999 [1]. At that point, the term was dedicated exclusively to the idea of assigning RFID tags to products that would be tracked. Nowadays, this paradigm encompasses a wide range of application domains, including smart homes [2], agriculture [3], industry [4], and smart cities [5]. IoT transforms regular objects and sensors in Internet nodes, allowing them to interact with human beings and other machines to carry out their tasks. As a consequence, the perspective of having ubiquitous and pervasive computing devices supporting our daily activities without significant human intervention has been increasingly consolidated [6,7].

Unlike conventional computers and smartphones, most IoT devices are not designed to provide Internet connectivity as one of their main functions. Even so, they collect and transmit lots of data of their surrounding environments, which is usually security-sensitive. They also can receive remote commands to act in multiple situations, including some critical ones. As these devices are not as secure as other computing devices but also get involved in security-sensitive tasks, they represent a perfect target for malicious activities.

Among several threats, botnets stand as one that can benefit the most from IoT security vulnerabilities. Botnets are networks made up of nodes that were infected by malware, which turned them into bots that can attack any target as a response to commands of a botmaster. Two main reasons make IoT an appropriate environment for botnets. First, the lack of security capabilities in IoT devices eases the installation and propagation of malware. Second, the huge number of IoT devices to be connected in next years provide the attackers with an unprecedented amount of vulnerable resources to support massive attacks. The denial of service attack performed by the Mirai botnet in 2016, which knocked down a major DNS provider in the United States, showed the destructive potential of this threat. In that occasion, the Mirai botnet generated peak traffic loads of 1.2 Tbps [8,9,10].

With those constant threats being developed and the lack of tools to increase the security of IoT devices, users can not rely only on updates for every security breach found. This scenario makes an intrusion detection system (IDS) fundamental for these devices [11]. Although the research community has proposed IDSs for IoT devices [12], they still present some open issues. The first issue is that several works such as [13,14,15,16] are mostly focused on protecting wireless sensor networks based on the 6LoWPAN protocol stack. These networks are an important part of the IoT universe, but many other kinds of IoT devices and networks also deserve attention, like the domestic devices. Relying on algorithms that require labelled data to be trained, as in [15,17,18], is another problem. Labelled samples of botnets attacks are difficult to obtain, and this data also usually becomes quickly outdated by new botnets and attacks. Lastly, various works, e.g., [19,20], explore network data for detecting attacks. It may be an issue since most of the communications are protected by end-to-end encryption, making it more difficult to obtain useful information from traffic data.

This work proposes the IoTDS (Internet of Things Detection System), an approach to detect botnets in IoT devices that aims to address the mentioned issues. The proposed approach makes use of one-class classifiers, which do not require labelled samples of malicious activities to train a model. One-class classifiers are a particular type of machine learning (ML) technique, which instead of classifying an instance in one of the multiple pre-defined patterns, they model a single pattern and use it to discern whether a new instance belongs to that pattern. IoT devices have specialised behaviour, performing simple tasks with a well-defined use of computational resources. We claim it is possible to model the device’s host-based data, more specifically CPU utilisation and temperature, memory consumption, and the number of running tasks, with a one-class classifier to support the detection of a botnet infection as an anomaly in the device’s behaviour. Therefore, the proposed solution is host-based and does not rely on network traffic data to detect the botnets. To avoid overloading its host, the approach is based on an agent-manager architecture that moves the effort of training a model from the host to a dedicated server. Finally, our approach was designed to detect botnets in more robust types of IoT devices, which usually have an operating system for constrained devices (e.g., Raspbian and Android) and at least 512 MB of RAM (Random Access Memory). These devices are found today in many homes and offices being used in diverse applications. Examples of this type of device are domestic routers, Raspberry Pi (https://www.raspberrypi.org/), Odroid (https://www.hardkernel.com/), Google’s Chromecast (https://store.google.com/product/chromecast) and Roku TV (https://www.roku.com/en-gb/).

To test our approach, we used the experimental setup created by [21]. The dataset contains data collected from an IoT device, a Raspberry Pi, which was infected by seven different botnets (Hajime, Aidra, BashLite, Mirai, Doflo, Tsunami, and Wroba). The Raspberry Pi emulated the behaviour of a surveillance camera and a multimedia centre. Four one-class classifiers were evaluated, namely Elliptic Envelope (EE), Isolation Forest (IF), Local Outlier Factor (LOF), and One-class Support Vector Machine (OSVM). In our tests, the proposed approach presented a high performance in detecting botnets, considering the different device profiles and the seven botnets, whilst it had a low impact in the host’s utilisation of CPU, memory and energy consumption.

This paper extends our previous work on detection of IoT botnets [22]. Unlike our previous work, which relied only on OSVM, in this work, we tested three additional algorithms. Besides, we proposed and implemented a novel agent-manager architecture based on HTTPS, detailing its building blocks and how they interact with each other.

This work is organised as follows. Section 2 presents the related work, while Section 3 introduces background information about one-class classifiers. In Section 4, the description of the proposed host-based detection approach is presented. Section 5 presents the results and the discussion about them. Lastly, Section 6 provides conclusions and future work.

## 2. Related Work

Most of the works on intrusion detection for IoT are based on simulated networks using the protocol 6LoWPAN [12]. The two most common operating systems deployed at these 6LoWPAN-based devices are Contiki and TinyOS. Although these works have promising results, exploring host-based, network-based and hybrid IDS, their focus on 6LoWPAN networks may prevent their application in networks based on other IoT protocols, such as Bluetooth Low Energy, and the conventional TCP/IP stack.

One of the most mature IDS for 6LoWPAN networks was dubbed SVELTE [13]. It is composed of several modules to protect devices from different attacks. The first module of the system uses the routing protocol RPL (Routing Protocol for Low power and Lossy Networks) to map the network topology and discover the nodes within the reach of IDS agents that are distributed over the network. Then, the distributed IDS agents check the packets of their neighbours. This process aims to protect the devices from insider attacks. Another module is a distributed firewall deployed on the devices, which protects them from an outsider attack, closing ports, and blocking addresses. SVELTE was tested in a network of Contiki-based devices.

In [14], an IDS is developed for TinyOS. The proposed IDS has some watchdog devices to analyse the packets being sent by their neighbours. This allows the system to detect traffic-based attacks and abnormal behaviour on the network. The IDS also enables the creation of rules and countermeasures that must be executed by the devices upon the detection of an intrusion. A limitation of their approach is that the rules may not have the needed complexity for responding accurately to a network attack, and the communication between the devices and the management module is insecure.

Granjal et al. [15] also proposed an IDS for 6LoWPAN networks, but the proposed system has a particular focus on the Constrained Application Protocol (CoAP), an application layer protocol for 6LoWPAN-based constrained devices. The proposed system is deployed in a centralised node in the network, which acts as a network sniffer to capture the packets within its network domain. To analyse the captured packets, the system first extracts different features from IEEE 802.15.4, 6LoWPAN, IPv6 and CoAP protocol headers. These features are selected using two methods, Principal Component Analysis (PCA) and Linear Discriminant Analysis (LDA). Then, Support Vector Machine (SVM), a supervised classification algorithm, is applied to classify the extracted data. To test the approach, the authors built an experimental setup on IoT-Lab (https://www.iot-lab.info/). As the authors employed SVM, they need samples from the different classes they modelled to induce their classifier, which can be an issue. Besides, the system relies only on network-based data, but they did not discuss how it would deal with encrypted traffic.

Other works on intrusion detection for IoT focus on conventional TCP/IP stack and IoT domestic devices. Most of these works are IDSs intended to be a module in a router or gateway, using whitelists, machine learning methods, or pre-defined rules. In [19], it is proposed an IDS named Heimdall. This IDS uses a whitelist to prevent IoT devices from connecting to malicious addresses, avoiding communications with botnets C&C (Command and Control) or private data leaks. Heimdall is intended to be a module in the local router, acting as a gateway for IoT devices, using third-party analysis of malicious addresses from organizations such as VirusTotal, Metadefender, and VirScan, combined with an auditor and DNS validation. They tested this approach on real domestic IoT devices, and the method is effective against the attacks developed by the researchers, as well as has minimal overhead. However, tests with real botnet attacks were not carried out, and the IDS depends on the maintenance of the third-party systems.

Other works such as [20,23] aim to build anomaly-based systems to detect IoT botnets. These works present techniques that model the legitimate behaviour of IoT devices. This type of approach can be useful due to the systematic behaviour of IoT devices, which usually perform specialised tasks. Thus, behaviour deviations are detected as malicious, regardless of the botnet.

In one of these works, Meidan et al. [20] proposed the N-BaIoT system, which uses deep autoencoders to detect botnets in IoT devices. They use the network traffic of IoT devices to build a model of legitimate behaviour and detect anomalies. The authors tested the approach on two botnets, Mirai and BashLite, and without using any labelled data, they achieved great results in detecting the attacks, with low false positives rate. Despite the great results, the use of deep autoencoders can be computationally costly even to a gateway and demands large amounts of data to train the model. The work proposed by [23] explores the construction of an IDS to protect Linux routers, a very popular target in recent years. They tested three different types of anomaly detection techniques, PCA, OSVM, and a naive detector using n-grams, to analyse syscall data from routers. They used two botnets to test their approach, MrBlack and Mirai, in simulated routers, and all tested methods presented good results. However, it can be challenging to find the best features among syscalls during data pre-processing, due to the large number of syscalls generated by multiple programs running in the system.

Overall, different issues were found in these works such as the lack of testing in real devices or with different botnet samples, dependency on third-party systems, use of computationally costly methods, and need for large amounts of data to train the models. Also, the analysed works are mostly network-based approaches and did not explore resource consumption data to detect botnets. Considering these gaps, we proposed an approach for botnet detection in IoT devices that do not need malicious labelled data to build a detection model and relies on host-based data such as CPU and memory utilisation. Additionally, our solution does not compromise the device’s performance or requirements since it is designed to be used in more robust IoT systems.

## 3. One-Class Classifiers

In traditional supervised classification techniques, data collected previously has to be associated with labels representing all the classes of interest when inducing a model capable of detecting each class. This requirement for labelled data from each possible class can be problematic in some cases, e.g., when the labelling cost is too high, or some class occurs much less frequently [24]. When considering botnet detection, labelled data may be collected by a specially designed testbed or honeypot. This requires manual work to set up vulnerable devices and some kind of access to botnets. Additionally, there is an inherited class unbalance problem when dealing with malware detection, where legitimate data is easily available and in a far greater scale.

An emerging alternative is the use of one-class classification techniques, which do not model all classes. Instead, data from a class of interest is used to create a model capable of yielding if a given new instance of data belongs to this class or not [24]. In the context of botnet detection, after collecting legitimate behaviour data (which is more straightforward to obtain than malicious data), one-class classification algorithms induce a model that discerns if a given new instance is legitimate or not. Here, it is considered that when an instance does not fit within the model boundaries, it is associated with malicious behaviour. In this sense, one-class classifiers can be used as an alternative to traditional supervised classification algorithms to reduce the labelling cost and the need for malicious data in the training phase significantly. Likewise, they are easier to adapt, given that periodical model updates using new legitimate data demand less effort than updates with labelled legitimate and malicious data. In this work, we compare the performance of 4 one-class classification algorithms: EE [25], IF [26], LOF [27], and OSVM [28].

These four algorithms were selected as they represent different approaches in one-class classification. The first algorithm, EE, was chosen due to its simplicity in detecting outliers. This algorithm computes an ellipse that separates the outliers from the inliers. Next, representing ensemble methods, we picked IF. Ensemble methods are reportedly robust and have been used in many network security problems [29], being a possible solution for this work due to its highly accurate predictive performance. We also wanted to have a clustering-based approach that could analyse the data using the similarity between the normal instances, thus we chose LOF. Lastly, we searched for an algorithm that has good results in building reliable models for detecting behaviour changes, and we found OSVM, that was successful in detecting machine faults [30].

### 3.1. Elliptic Envelope

EE [25] is a simple method to detect outliers based on a known distribution. The algorithm models the data as a high dimensional Gaussian distribution with possible covariances between the features [31]. Intuitively, the algorithm tries to find an ellipse that represents most of the common behaviour data. The data inside the ellipse is considered inlier, and the outside points are outliers. To estimate the size and the shape of the ellipse, EE relies on the Fast Minimum Covariance Determinant (FAST-MCD) method [25]. FAST-MCD uses the Mahalanobis distance, which is a measure of the distance between a point *p* and a distribution *D*, in conjunction with a covariance matrix to fit this ellipse. The hyperparameter of this algorithm is the contamination, which indicates the fraction of points in the data the algorithm can discard to fit the shape. This is used since the presence of outliers in the data can cause some distortion on the fitted Gaussian.

### 3.2. Isolation Forest

IF [26] builds decision trees to detect anomalies, instead of profiling common data points. The partitions made by decision trees are created by randomly selecting a feature and then selecting a random split value between the minimum and maximum value for this feature in the training dataset.

The main idea of this process is that outliers are atypical instances that do not follow the pattern of the majority (common behaviour) of the data. The random partitioning will put these outliers closer to the root of the tree, creating trees with shorter path length. Thus, if an instance is in a shorter path of a tree, it is an outlier. In this method, an anomaly score represented by the following equation is used:

(1) $$s\left(x,n\right)=2^{-\frac{E(h(x))}{c(n)}},$$

where h(x) is the path length of an instance *x*, c(n) is the average path length of a unsuccessful search in the binary tree, *n* is the number of external nodes and E(h(x)) is the average of h(x) from a collection of isolation forest trees [26]. Every observation receives a score. If s(x,n) is close to 1, this indicates an anomaly. A score smaller than 0.5 indicates a normal instance.

### 3.3. Local Outlier Factor

LOF estimates a score, named Outlier Factor, which reflects the level of abnormality of each observation from a dataset [27]. It works based on the idea of a local density. The *k*-Nearest Neighbours algorithm is applied in the data, and each data instance is given a locality, which is used to estimate the clusters’ density. First, the number of neighbours must be decided using a hyperparameter *k*. Choosing an appropriate value for *k* is very important, since a small *k* has a tighter focus, but has more errors when dealing with noisy data. On the other hand, a large *k* can include all the instances that are outliers.

The algorithm uses a function called *k-distance*, which is the distance of an instance to its *k*-th neighbour. This *k-distance* is used for the calculation of the reachability distance (RD), which is the largest value out of the distance between two instances and the *k-distance* of the second instance, as showed on Equation (2). If an instance *a* is in the *k* neighbours of *b*, RD(a,b) is equal to the *k-distance* of *b*, otherwise it will be the real distance between *a* and *b*.

(2) RD(a,b)=max{k_distance(b),distance(a,b)}

This RD function is used to calculate another function, the local reachability density (LRD). To calculate the LRD of an instance *a*, the RD of *a* to all its *k* neighbours ($N_{k}\left(a\right)$) is calculated at first, followed by the average of the resulting values as presented on Equation (3).

(3) $$LRD\left(a\right)={\left(\sum_{n}^{N_{k}\left(a\right)}\frac{RD(a,n)}{k}\right)}^{-1}$$

Next, the LRD of each point in the dataset is calculated. Each point compares its LRD to their *k* neighbours’. The LOF is the average ratio of the LRD of a point *a* to the LRDs of its closest points, *k* neighbours. As shown on Equation (4), if the LOF is greater than 1, the LRD of a point *a* is on average greater than the LRD of its *k* neighbours.

(4) $$LOF\left(a,N_{k}\left(a\right)\right)=\left\{\begin{array}{cc} \approx 1 & \mathrm{Similar}\;\mathrm{density}\;\mathrm{as}\;\mathrm{neighbours}\\ >1 & \mathrm{Lower}\;\mathrm{density}\;\mathrm{than}\;\mathrm{neighbours}\;\left(\mathrm{Outlier}\right)\\ <1 & \mathrm{Higher}\;\mathrm{density}\;\mathrm{than}\;\mathrm{neighbours}\;\left(\mathrm{Inlier}\right) \end{array}\right.$$

### 3.4. One-Class Support Vector Machine

OSVM is an extension of the SVM created by Cortes and Vapnik [28], which is a traditional supervised ML algorithm used for classification problems. The algorithm works by creating a hyperplane (*n*-dimensional plane) that better separates two different classes. SVM tries to optimise the separability of the data, searching for instances that are within the boundaries that split the data. Those instances are called Support Vectors. For the use of SVM, we need to process the data in kernel functions, which are set through a hyperparameter. The kernel functions are responsible for taking non-linearly separable data and implicitly mapping them to a higher dimensional plane that can be linearly separated.

Unlike the traditional SVM, OSVM uses the hyperplanes to create boundaries around a region that better contains all training data. By doing so, OSVM is capable of identifying if an instance is inside the area or not. To create these hyperplanes, the OSVM algorithm optimises the following objective function:

(5) $$\min_{w,\xi ,\rho }\frac{1}{2}w^{T}w+\frac{1}{\nu N}\sum_{i=1}^{N}\xi i-\rho$$

subjected to $w^{T}\phi \left(x_{i}\right)\geq \rho -\xi_{i}$ and $\xi_{i}\geq 0$, where $x_{i}\in R^{p}$ is an instance of the training set, *w* is the weight vector of the hyperplane in the inner product space, ϕ(.) is a mapping from the original *p*-dimensional feature space to an inner product space, $\xi_{i}$’s are penalty terms for error, ρ is a bias term, and ν∈(0,1] is a parameter that poses an upper bound on the fraction of outliers in the training set.

The decision hyperplane can be represented by $g\left(x\right)\equiv w^{T}\phi \left(x\right)-\rho =0$. The solution of the convex optimisation problem of the objective function finds the decision hyperplane, and the corresponding decision function f(x):

(6) $$f\left(x\right)=\left\{\begin{array}{cc} w^{T}\phi \left(x\right)-\rho \geq 0 & \mathrm{if}\;x\;\mathrm{belongs}\;\mathrm{to}\;\mathrm{the}\;\mathrm{set}\\ w^{T}\phi \left(x\right)-\rho <0 & \mathrm{if}\;x\;\mathrm{is}\;\mathrm{an}\;\mathrm{outlier} \end{array}\right.$$

In this work, it is used the radial basis function (RBF), the polynomial and the linear kernel to map the input space to a higher dimensional space. The RBF kernel is described by the following equation:

(7) $$\phi \left(x\right)=\exp(-\gamma ||x-x^{\prime }|{|}^{2}),\mathrm{where}\;\gamma >0.$$

The hyperparameter γ is the influence in the training samples. Next, the polynomial kernel is described by the equation:

(8) $$\phi \left(x\right)={\left(\gamma \langle x,x^{\prime }\rangle \right)}^{d},$$

where *d* it is the degree function passed as a hyperparameter. Lastly, the linear kernel is represented by the equation:

(9) $$\phi \left(x\right)=\langle x,x^{\prime }\rangle .$$

## 4. IoTDS: A Host-Based Botnet Detection System for IoT

In this section, the IoTDS is described. Firstly, a system overview and the threat model are presented. Next, the architecture is introduced, detailing its two main components, Agent and Management Console. Lastly, the two phases of the detection process, Model Induction, and Continuous Analysis are explained.

### 4.1. IoTDS Overview

Since IoT devices are very specialised, running simple, repetitive, and well-defined tasks, these devices behaviour are supposed to present a precise level of regularity as long as they are not compromised. Likewise, malicious software that compromises an IoT device should alter its behaviour. Therefore, it is possible to take advantage of a one-class classifier to model the device’s behaviour and detect anomalous events, avoiding the effort demanded by collecting and labelling malicious data to train the behaviour model.

The IoTDS is a host-based system. Running the system in an IoT device, instead of at some point of the network, allows the collection of data about the device’s resource consumption, which can be more sensitive to the presence of bot malware. Also, by relying only on resource consumption data, the IoTDS ability to detect malicious activity is not hindered by the encryption of malicious traffic. Lastly, IoTDS can be pre-installed in IoT devices, offering protection to these devices out of the box.

The IoTDS works creating models of the legitimate behaviour of the IoT devices and detecting any anomalies using host-based data. If the behaviour of the device presents an anomaly, the device sends an alert to a central server. After a given number of consecutive alerts, the system tries to stop the unusual behaviour executing a reboot of the IoT device’s system automatically.

The IoTDS is divided into two phases, Model Induction, and Continuous Analysis. These two phases are executed for each IoT device. Also, the system architecture is divided into two elements: Agent, installed in the IoT device and responsible for analysing the device’s data, and Management Console, installed in a separated computer server, where the alerts are stored, and an overview of the situation of the device is provided to the network administrator.

Two main challenges coming from our design choices were addressed in this work. Being a host-based solution, the system can not consume a large amount of the device’s resources, since that would be harmful to its other functionalities. Bearing this in mind, we move the heaviness of inducing a model to a centralised server with more computational power, which hosts the IoTDS Management Console. Hence, the IoT device runs an already trained behaviour model, which can be done at a minimum cost of computational power. Besides, the chosen features can be easily extracted, which avoids an overhead related to this task.

Processing data in a separated computer brings challenges regarding channel security to the proposed system. Since the IoTDS Agent captures host-data about the device, it is necessary to build a secure communication between the Agents and the Management Console. In the proposed approach, the HTTPS protocol is used to protect the information travelling between the Agents and the Management Console. HTTPS provides privacy and integrity, as well as allows the authentication of the end points [32].

The IoTDS is designed to offer the following advantages:

1. *Distributed architecture*: although a main server is responsible for inducing the behaviour model, the continuous classification of new observations is performed by distributed agents deployed in the hosts. Therefore, the server does not have to analyse data coming constantly from a large number of IoT devices. This saves network resources and improves system scalability;
2. *No need for Internet connection*: the system does not need any Internet connection or use third-party online software to detect IoT botnets, such as whitelists and other software to verify valid IP addresses/ports;
3. *No use of malicious data during the training step*: the system does not need the collection or use of malicious data to induce the classification model during the training step. It only has to capture legitimate data on the IoT device’s behaviour to do it;
4. *No use of traffic analysis*: the system does not analyse traffic data. When botnet traffic is encrypted end-to-end, network-based solutions can face difficulties to analyse the traffic.

### 4.2. Threat Model

Our threat model is based on the typical behaviour of IoT botnets such as Mirai. These botnets are usually composed of the following elements: bots, C&C server, scanner, reporting server, loader, and malware distribution server. As already mentioned in this work, bots are compromised devices that are used to carry out powerful attacks against selected targets. The C&C server is the central console the attacker uses to control the entire botnet operation. The scanner is a software agent responsible for scanning the Internet looking for devices that are potential bots. These devices have open ports that can be accessed through the Internet and some well-known vulnerability. Devices that can be accessed through Telnet using default credentials are a typical example. When the scanner finds a device that matches the desired characteristics, it sends the device’s basic information to the reporting server, which stores it. The loader uses the information in the reporting server to log into the vulnerable devices. Then, it makes the vulnerable devices download the malware code from the malware distribution server. The malware is installed in the device’s RAM, and its downloaded binary is deleted. From this point on, the malware keeps listening to a device’s port, waiting for commands from the C&C server [8,10,33].

IoT botnets are known by the simplicity of their operation. Based on our observation of IoT botnet malware functioning and literature reports [8,10,33], we assume that they can try to kill competing malware at the same device and other processes that use the same ports as them. However, they do not bring other legitimate applications down. Moreover, as they are installed in the device’s RAM, they do not persist after the device’s reboots. Installing the malware only in RAM sounds as if attackers made a design mistake, but it is a matter of choice. IoT botnet malware is designed to be very aggressive at scanning, being able to recruit many bots quickly. Therefore, they do not need to put a great effort in persisting in the devices.

### 4.3. IoTDS Architecture

**Figure 1:**
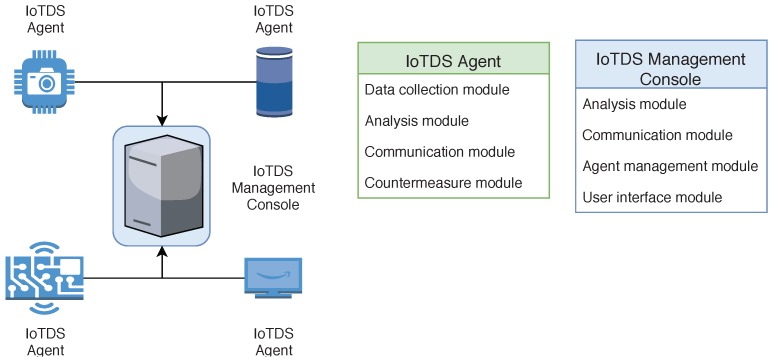
IoTDS architecture overview.

The approach is divided into two elements: IoTDS Agent and IoTDS Management Console. As presented in Figure 1, the Agent is installed in the IoT devices, and the Management Consoled in a separated computer connected to these devices. Next, more details about these two elements are provided.

#### 4.3.1. IoTDS Agent

The IoTDS Agent is installed in the IoT devices, and it is responsible for capturing the device’s data, analysing it, and sending alerts to the Management Console when necessary. This element has the following modules: Data Collection Module, Analysis Module, Communication Module, and Countermeasure Module. The Data Collection Module collects the following descriptive data about the system behaviour: CPU and memory utilisation, number of running tasks, and CPU temperature. This module is used in both the Model Induction and Continuous Analysis phases.

The data collected by this module is processed to be used by the Analysis Module or sent by the Communication Module to the IoTDS Management Console. How data is processed depends on the purpose of its collection. During the Model Induction Phase, the data is collected each second during an interval of *y* seconds, stored, and then sent to the Management Console, which will build the model. In the Continuous Analysis phase, this data is consumed by the Analysis Module to be classified.

The Analysis Module uses a model sent by the IoTDS Management Console to analyse data collected in real time and determine whether an anomaly is occurring. The Communication Module is responsible for the communication between the Agent and the Management Console, sending collected data and alerts, as well as establishing a secure channel for this communication. Finally, the Countermeasure Module is responsible for rebooting the system when it receives the system’s reboot command.

#### 4.3.2. IoTDS Management Console

The IoTDS Management Console is responsible for creating behaviour models for the IoTDS Agents, storing alerts and information, and presenting alerts and information about the IoT devices to the network administrator. The Management Console is installed in a separated server connected with the IoTDS Agents. It can be installed in the gateway of an IoT network, which has access to all devices and enough computational power to run all the Management Console’s modules. Also, the IoTDS Management Console can be installed in a local desktop computer connected to the private network. The modules that compose the Management Console are the Analysis Module, the Communication Module, the Agent Management Module, and the User Interface Module. The Analysis Module is responsible for creating behaviour models for the IoT devices.

Since IoT devices have constrained computation power, they cannot build a behaviour model without compromising their functionalities and battery power since this is a computationally heavy task. Also, in a centralised server, it is quicker to build a behaviour model, and it is easier to change the behaviour model in use. The Agent Management Module has the functionality of registering the active IoTDS Agents, their alerts, and their information. This module works when an Agent sends a request for a behaviour model. Then, the Agent Management Module stores its IP address and the device’s name. Also, this module is called when an Agent sends an alert. The Communication Module has the same responsibilities as its homonym in the IoTDS Agent. The IoTDS Management Console creates an HTTPS based channel, which offers integrity, privacy, and trust. The adoption of HTTPS also prevents replay attacks, meaning that an attacker cannot, for example, take a reboot command and send it again to the IoTDS Agent. Finally, the User Interface Module is responsible for presenting information about the generated alerts and active agents to the network administrator.

### 4.4. Process Details

This section presents the details on how the IoTDS carries out botnet detection. This process is divided into two phases, the Model Induction, and the Continuous Analysis phases.

#### 4.4.1. Model Induction Phase

**Figure 2:**
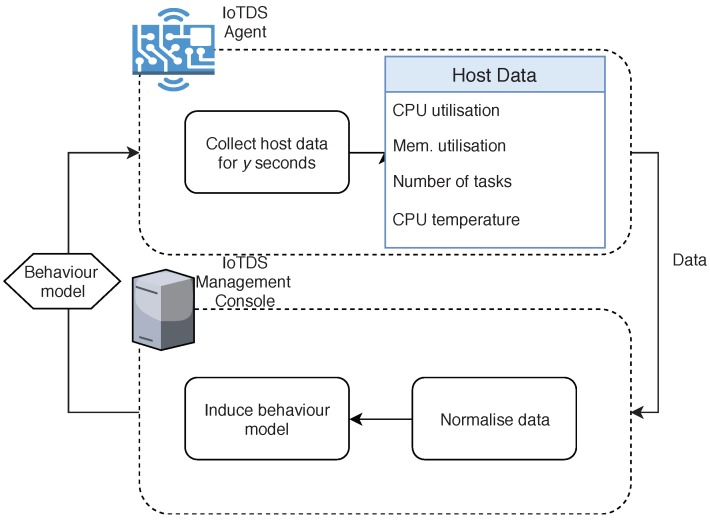
Description of the Model Induction phase.

The Model Induction phase, illustrated in Figure 2, consists of gathering host’s data and building a model. First, the IoTDS Agent collects the memory and CPU utilisation, the number of running tasks and the CPU temperature at every second for a training interval of *y* seconds. Then, the data instances are extracted from these observations according to an *s*-second time-window to induce the behaviour model. The value $x_{i}\left[j\right]$ of a data instance *i* for a particular feature *j* will be the arithmetic mean of the observations collected for *j* during the *i*-th *s*-second time-window. All the extracted data instances for the *y*-second training interval are included in a CSV file, which is sent to the IoTDS Management Console in an HTTP-POST request through an HTTPS-based connection.

Then, in the Management Console, all features for each instance are scaled according to their minimum and maximum values between a range from 0 to 1, defined by the following equation:

(10) $$x_{i}\left[j\right]=\frac{x_{i}\left[j\right]-min\left(x\left[j\right]\right)}{max(x[j])-min(x[j])},\forall i\in I,\forall j\in J$$

where *i* corresponds to a given instance, *I* to all instances collected during that period, *j* to a feature in the *J* feature space, and $x_{i}\left[j\right]$ to the value present in the feature *j* for the instance *i*.

Once the instances are normalised, the Management Console induces the behaviour model, which is stored in a file and sent in response to the IoTDS Agent’s previous request for the model.

#### 4.4.2. Continuous Analysis Phase

**Figure 3:**
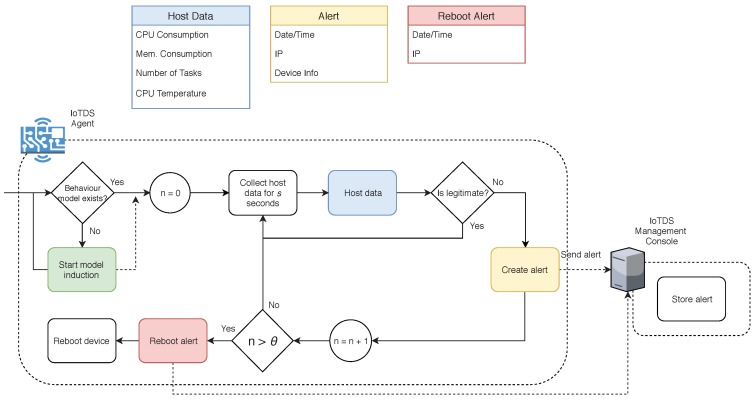
Description of the Continuous Analysis phase.

The Continuous Analysis phase, presented in Figure 3, consists of using the induced model to monitor the device and try to prevent IoT botnets from running in the system. The IoTDS Agent first checks whether there is a behaviour model in the system. If the Agent does not have a behaviour model induced by the Management Console, it starts the Induction Model phase. After this check, the system starts a trust factor counter *n* with zero. Next, the Agent collects the observations to produce a new data instance according to the *s*-second time-window used to induce the behaviour model. This new instance undergoes the same normalisation step described in Equation (10), except that this time, the max(x[j]) and min(x[j]) are the same as the ones used when the behaviour model was induced. After that, the approach decides whether this new instance is a legitimate behaviour or an anomaly. If it is the latter, the device raises an alert.

The alert is sent to the IoTDS Management Console with information about the device’s IP address, information about the resources utilisation and the timestamp of the alert. Once the IoTDS Management Console receives the alert, it is stored and presented in the user interface for the network administrator analysis. After the alert is sent, the IoTDS Agent increments the trust factor *n* by one and resumes the host data analysis.

The objective of the trust factor is to avoid generating an excessive number of alerts during an infection. If a device is compromised, the resource consumption data changes its behaviour for as long as the malware operates. Consequently, the Agent detects this unusual behaviour every time it analyses the devices’ data, generating an alert at every new iteration. Then, the trust factor takes place to control this situation. Most of IoT botnets, such as Mirai, BashLite, and Hajime run in the device’s RAM and, as a consequence, are not persistent. Thus, a system reboot is effective in removing the malware. When the trust factor reaches θ, a threshold that can be set by the network administrator, the IoTDS Agent sends a reboot warning to the IoTDS Management Console informing the device’s IP address and the timestamp, and then it reboots the device. When the device restarts, the Agent resumes the Continuous Analysis phase.

## 5. Results and Discussion

In this section, we provide the results of the proposed approach. First, we present the experimental environment and the technologies used to implement the system. Then, we divide the evaluation into two parts. The first one is related to the predictive performance of our approach. In this part, we execute the system with the one-class algorithms discussed in Section 3. Our objective is to check which algorithms have the best performances and whether at least one of them can show a high detection rate along with a low false positive rate. In the second part, the impact of the system in the IoT device is studied. CPU and memory utilisation, CPU temperature, and energy consumption are observed to analyse how much impact the system has on its host’s main resources.

### 5.1. Experimental Environment

**Figure 4:**
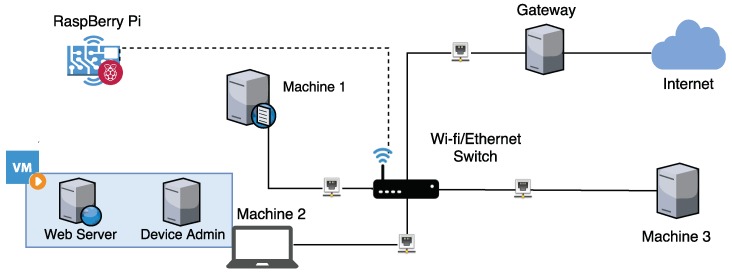
Network topology of the experimental environment.

To test the proposed approach, we used the experimental environment built by [21], which is illustrated in Figure 4. The network components consist of a switch with Ethernet and Wi-Fi interfaces, and four computers (Machine 1, Machine 2, Machine 3, and Gateway) connected to the switch through Ethernet. There is also a Raspberry Pi model 3B connected to the switch through the Wi-Fi interface. The Machine 2 provided two virtual machines, with the first one hosting a Web Server and the second being a client, referred to as Device Admin.

The Raspberry Pi emulates three different behaviour profiles: two focused on security cameras and one focus on a multimedia device.

- Multimedia Centre (MC): found in today’s living rooms, a multimedia centre is a device that consumes streams of video such as movies or TV shows, to transform regular TVs in smart TVs. In addition to the use of video content, it also accesses an application store for updates and installation of other programs. The task of rendering video combined with the heavy traffic generated by video streams make this device to use a significant portion of its resources during its operation;
- Surveillance Camera with Additional Traffic (ST): used both on the inside and outside parts of houses and companies, the operation of surveillance cameras is mostly characterised by the transmission of a video stream to a computer or station. However, this kind of device can also present traffic from other protocols. Telnet or SSH connections can be established to check if the device is working, its temperature, and the running processes, for example. These cameras may also include configuration web pages, which users access from their browsers to set up the parameters of cameras operation. As the device do not have to render video before transmitting, it consumes fewer resources than the multimedia centre;
- Surveillance Camera (SC): this profile is similar to the ST profile, but it does not include interactions with users through Telnet, SSH, and configuration web pages. Having the video transmission as their single task, devices of this profile consume fewer resources than those of the ST profile.

The Gateway machine was used to provide Internet access and DHCP functionality to the network. The Machine 1 hosted a DNS server that was used by the Mirai botnet. The same machine also hosted a VLC client that consumed the video stream generated by SC/ST profiles and a VLC server that generated a video stream for the MC profile. The Machine 2 hosted the Web Server and the Device Admin. The Web Server was responsible for providing a static Web page, which was accessed by the Raspberry Pi in all the profiles to emulate the consumption of web services. The Device Admin emulated the transactions of an administration software used to control and set up the emulated camera in the ST profile. To do so, it interacted with the Raspberry Pi through Telnet, SSH, and the Web configuration page. Lastly, the Machine 3 was responsible for emulating an attacker in the network, infecting IoT devices with botnet samples. Particularly for Mirai, this machine also hosted a C&C server, which could be used to make the Raspberry Pi launch DoS attacks against selected targets.

We executed the following botnets in our experimental network: Mirai and Bashlite, the most famous IoT botnets that uses Telnet [10]; Hajime, a botnet of unknown purpose that patches vulnerabilities in IoT devices [34]; Aidra, Tsunami and Dofloo, very famous botnets that moved from personal computers domain to IoT devices using ARM processors [35].

With those botnets, we have three types of infection, as presented in Table 1. Legitimate data was captured following the description of each behaviour. The MC profile received video streams from YouTube, Twitch and HD video, while the SC device streamed video to another computer. The ST device streamed video, was accessed via SSH and Telnet and performed updates in programs. We captured three hours of legitimate data from each device profile, and captured one hour of each infection on each device, erasing the data in the device after each capture to prevent contamination from the previous botnet.

### 5.2. System Implementation

In this section, we describe how the IoTDS was implemented for the tests. The entire system was implemented in Python 3. The one-class classifiers were implemented using the Python Scikit-learn library 0.20 [36]. The Agent uses the Linux top program, which presents the utilisation of CPU/memory and the number of running tasks. This program is available in some devices’ OS and in busybox, which is present in surveillance cameras. The Raspberry Pi’s program vcgen was also used to collect information about the CPU temperature.

The communication between the Agent and the Management Console was made by using the library Python Requests (http://docs.python-requests.org/en/master/), which allows the communication between HTTPS endpoints. The IoTDS Management Console was built using the framework Flask (http://flask.pocoo.org/). The Python Joblib (https://joblib.readthedocs.io/en/latest/) library was used to export the induced behaviour models to the Agents. All the alerts are sent as HTTPS forms to the Management Console, and the reboot command was the one commonly used for Linux systems.

### 5.3. Predictive Performance

In this section, we evaluate the IoTDS’s predictive performance. The first part describes the tests setup and the second part the results of the algorithms.

#### 5.3.1. Experimental Setup

To evaluate our proposed approach, we divided the capture files in nine datasets, which are composed of three hours of legitimate traffic and one hour of malicious traffic each. Each dataset refers to a different profile and infection type combination, as presented in Table 1. We organised the evaluation into three phases: finding an optimal time-window size, considering that smaller windows will result in faster identification of botnets; discovering the least number of instances needed for training; and optimising the classifier’s hyperparameters. Table 2 presents the features and their respective value ranges in the datasets.

All tests were performed using the holdout method with 50 repetitions to minimise variance in the results. The metrics used to evaluate the proposed approach were [37]:

- **Precision**: $\frac{TP}{TP+FP}$ the percentage of classified botnets instances that are truly botnets;
- **Recall**: $\frac{TP}{TP+FN}$ effectiveness of the approach in identifying botnets;
- **Specificity**: $\frac{TN}{FP+TN}$ how effective is the approach in identifying instances that are legitimate;
- **Accuracy**: $\frac{TP+TN}{TP+FN+FP+TN}$ the overall effectiveness of the approach;
- **AUC**: $\frac{1}{2}\left(\frac{TP}{TP+FN}+\frac{TN}{TN+FP}\right)$ ability of the approach to avoid false classification;
- **F1-score**: $\frac{2}{\frac{1}{recall}+\frac{1}{precision}}$ relation between recall and precision.

TP, TN, FP and FN stand for true positives, true negatives, false positives and false negatives, respectively.

#### 5.3.2. Classifiers’ Performance

In this section, we discuss the experimental results of the tested algorithms: EE, IF, LOF, and OSVM. First, we used the default hyperparameters for the classifiers to analyse the impact on the predictive performance of changing the time-window.

Table 3 presents the average accuracy, precision, recall, F1-score, specificity, and AUC in all datasets for each classifier varying the time-window size. The best result for each metric and classifier is highlighted. It is possible to see that the LOF algorithm had the best results, followed by OSVM, IF, and EE considering the F1-score. Although precision is around 70% in the LOF classifier, the other metrics present satisfactory results. Still bearing the F1-score in mind, the best time-window size for this algorithm was 30 s, followed by 1 s with a small difference between them. Then, we chose the 1-s one. Although it is not the best time-window, by choosing the 1-s one, botnets would take less time to be detected.

After having defined the best time-window, we evaluated the amount of legitimate data needed to yield good predictive performance. First, we fixed the time-window to 1 s and used the default hyperparameters for each classifier. Then, samples ranging between 10% and 90% (10-point step) of the legitimate data were extracted to make the training dataset. In other words, at each iteration, for each profile-infection combination, we sampled a percentage of all legitimate data and induced the behaviour models on it. After that, the classifiers were tested using the remaining legitimate data and all botnet data. All performance metrics presented in Table 4 had very similar results, showing that the classifiers can use the smallest tested fraction of legitimate data to model legitimate behaviour and still reach high predictive performance. This also indicates that all legitimate data are very similar. Thus, the sample size of 0.1 (10%) was chosen.

**Figure 5:**
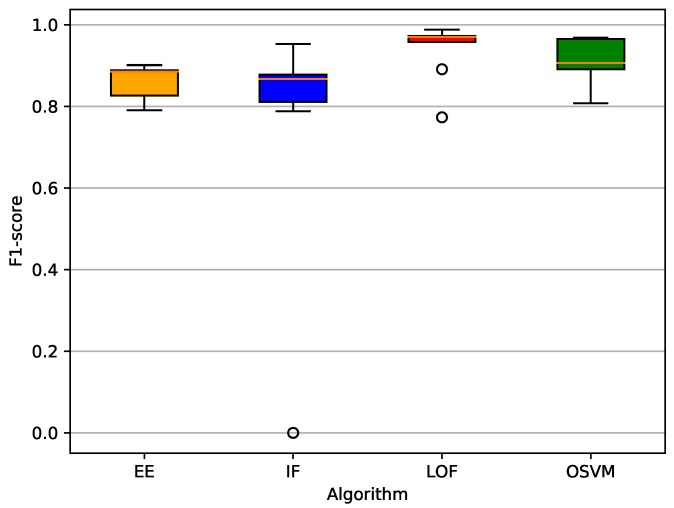
F1-scores considering all datasets with optimised parameters.

**Figure 6:**
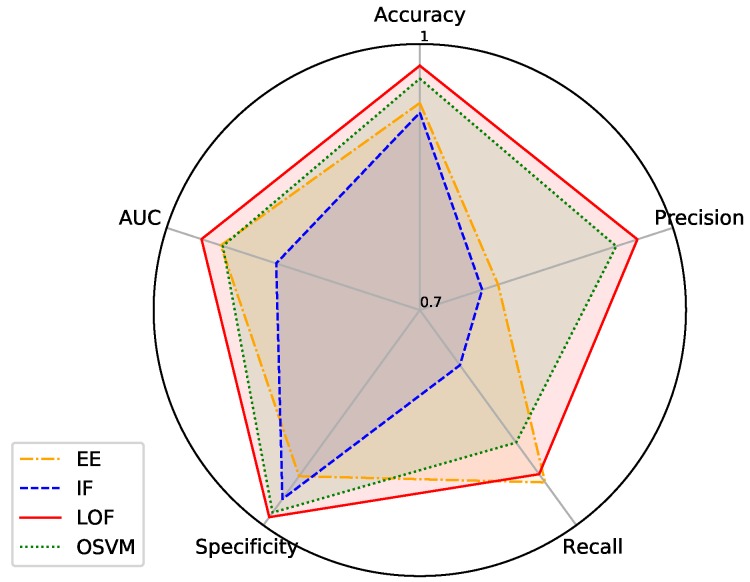
Mean results for accuracy, precision, recall, specificity and AUC considering all datasets with optimised hyperparameters.

After selecting the time-window and sample size, an optimisation of the classifiers’ hyperparameters was performed. We used a random search, a lightweight and effective method [38], to find the best combination of hyperparameters, as presented in Table 5. All the algorithms were submitted to tests to evaluate their best hyperparameters in the same normalised dataset, except for IF, which was not submitted to normalised data because it is a tree ensemble method that can deal with not normalised data. In our evaluation, we used the F1-score to find the best hyperparameters in each algorithm. The optimised hyperparameters for each algorithm are presented in Table 6. Figure 5 presents a boxplot of the F1-scores when using a 1-s time-window, a training sample size corresponding to 10% of legitimate data, and the optmised hyperparameters. Figure 6 shows the mean results for all metrics (except F1-score) also considering a 1-s time-window, a training sample size corresponding to 10% of legitimate data, and the optmised hyperparameters. Both plots aim to ease the comparison of the results for the different classifiers. F1-scores were placed in an exclusive plot because they can represent the overall performance of each classifier.

Since each dataset is composed of 10,800 legitimate and 3600 botnet instances, only 1080 (i.e., 10%) legitimate instances were used for training in each technique. Mean values for specificity and AUC were similarly good for all algorithms. On the other hand, precision and recall presented some variations for different algorithms, which demands more detailed analysis and can allow to point out which algorithms had better performances.

The first algorithm analysed is EE. This classifier presents a high performance on specificity and AUC, but is among the worst results for precision. The worst scenario of this algorithm was the Mirai botnet in the ST profile, and the best results were in the SC profile with Aidra. The high performance on the SC profile can be explained by the lack of additional legitimate interventions from users. Its behaviour is only based on its main function, which makes it easier to induce a proper model since the normal behaviour is more predictable. The ST profile, on the contrary, has more variations in its normal behaviour, meaning that it is more challenging to the classifier.

IF presented high values of specificity and AUC, though the precision and recall values do not support the use of this method. A clear example of this was the Mirai infection (I2) in the profile ST. In this dataset, the IF algorithm considers all the instances as legitimate, having a high specificity performance, but both precision and recall presented values of 0%. The ST profile includes some legitimate interventions on top of its main function of transmitting video, which induced the algorithm to classify the changes caused by Mirai as legitimate. The other datasets presented better results, being the profile SC infected by Mirai the one with the best outcomes.

The LOF algorithm had the best average results in all datasets, outperforming the OSVM classifier. The scenario with the best results for this algorithm was the infection type 1 (Aidra) on the SC profile with almost 99% of AUC. Also, the precision and recall presented a better performance than OSVM. The worst results of this algorithm were for the infection type 1 (Bashlite) on the ST profile with a recall of 65%. This may be a consequence of the ST profile’s peaks of usage, which made the algorithm classify legitimate variations as botnet behaviour. Nevertheless, the precision remained with a high score.

Lastly, OSVM had the second best results of the four algorithms, getting satisfactory results for precision and recall. The scenario with the best results for this classifier was in the SC profile with the Aidra botnet, and the worst was the same profile with the infection type 3 (Mirai, Doflo, Tsunami, and Wroba). This worst scenario presented a recall value of 76%, while all other datasets presented better results. This may indicate that, for this specific dataset, the selected hyperparameters were not the best, since they were selected considering all scenarios.

### 5.4. System Impact on Its Host

**Figure 7:**
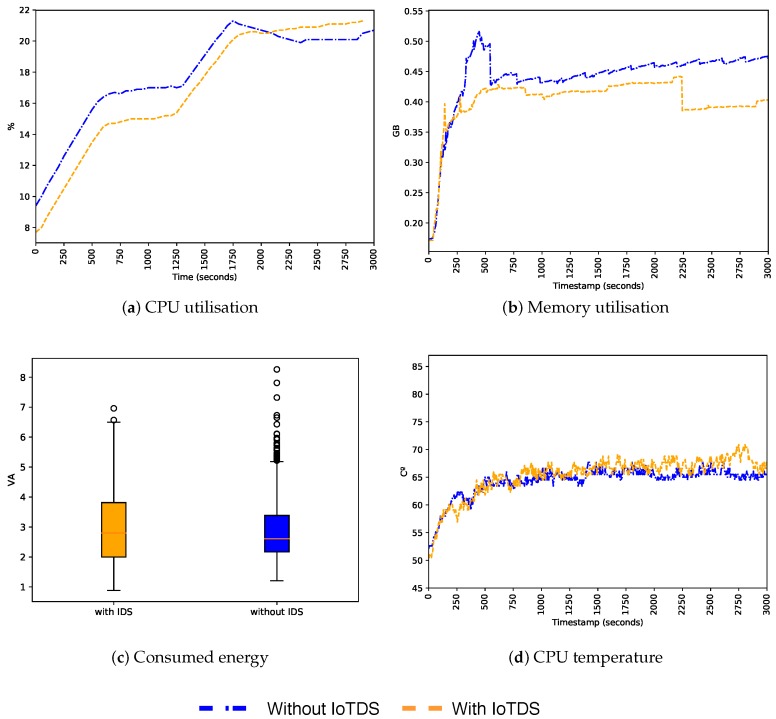
Behaviour of the MC profile regarding host’s resource usage with and without the IoTDS.

**Figure 8:**
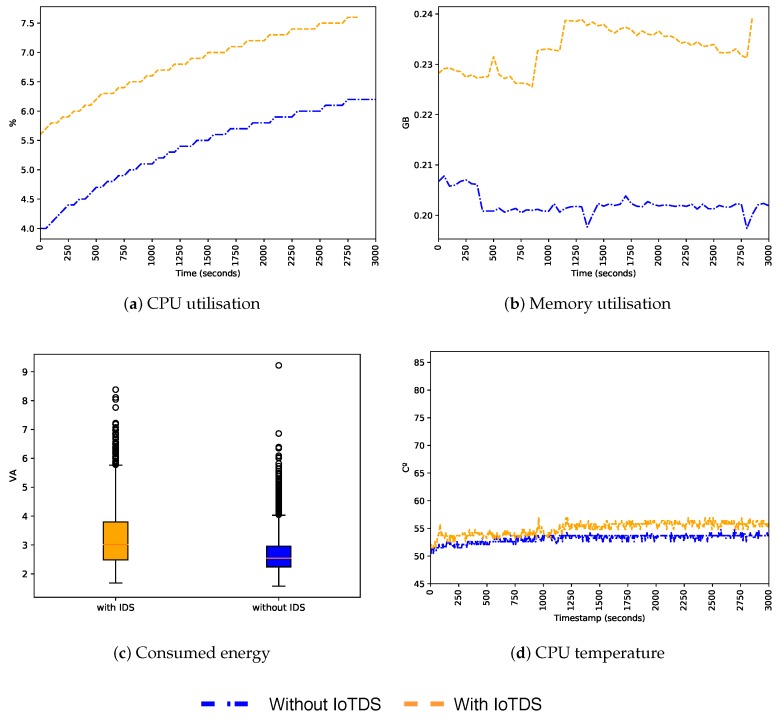
Behaviour of the SC profile regarding host’s resource usage with and without the IoTDS.

**Figure 9:**
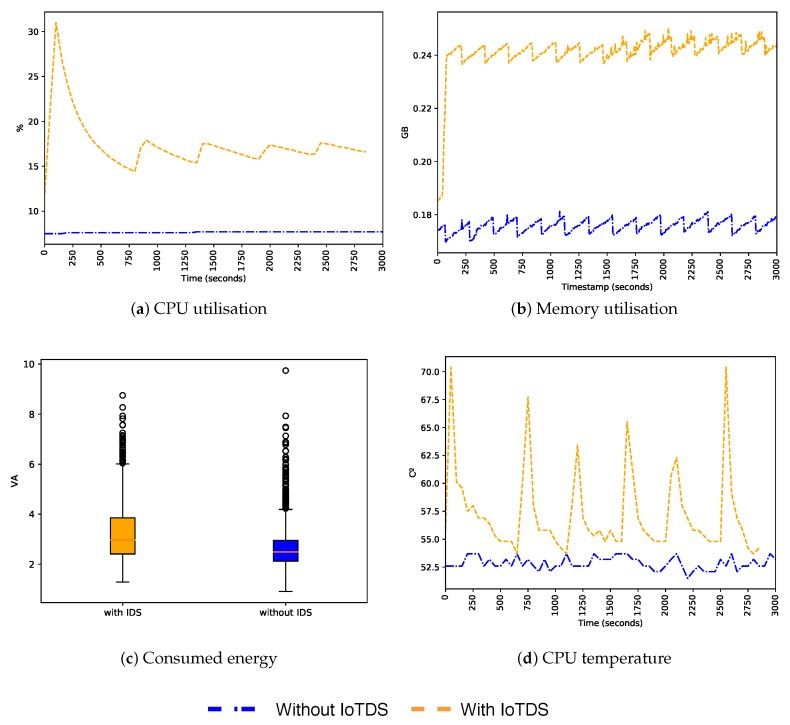
Behaviour of the ST profile regarding host’s resource usage with and without the IoTDS.

After evaluating the predictive performance of our approach, we also need to assess its impact when running in an IoT device. Since we built a host-based approach, and IoT devices have limited resources, it is essential that the resource consumption of IoTDS does not harm the performance of the device. To address this, we computed the device’s CPU and memory utilisation, energy consumption, and CPU temperature with and without the IoTDS running. Each monitored parameter has, respectively, the following minimum and maximum values: 0% to 100%, 0 GB to 1 GB, 1 VA to 10 VA and 0 °C to 100 °C. In Figure 7, Figure 8 and Figure 9, these values, for each IoT device profile, are presented. The blue line represents values computed when the IoTDS was not running, whereas orange lines comprehend to values collected with the IoTDS running. All the tests were performed with LOF using its optimised hyperparameters, as in Table 6.

In Figure 7, the MC profile is considered. The IoTDS seems not to have affected the device since the behaviours in the two scenarios are very similar. In this sense, the IoTDS did not compromise the device performance. The CPU and memory utilisation, as well as the CPU temperature, behave similarly. The energy consumption in the scenario was slightly higher on average.

Figure 8 presents the results regarding the SC profile. Among the three profiles, this one has the lowest level of resource use under normal conditions, so the impact of the IoTDS is more evident here. The CPU utilisation increased by two percentage points, followed by the CPU temperature that was around 5 °C higher. The memory utilisation increased by 40 MB but remained constant throughout time. This profile presented the same increase in energy consumption, as seen in the MC profile. Despite these increases, resources were far from being exhausted, showing that the IoTDS did not compromise the device operation.

Lastly, Figure 9 shows the results for the ST profile. Its behaviour was similar to the SC profile. When this profile was running the IoTDS, the CPU utilisation had a peak of 30%, quickly decreasing later and then, stabilising around 20%. The initialisation of the IoTDS may have caused this peak. The energy consumption also increased, having similar results as the MC and SC profiles. In the same manner, as in other profiles, the increase in resource consumption caused by the IoTDS did not seem to impair the device.

**Figure 10:**
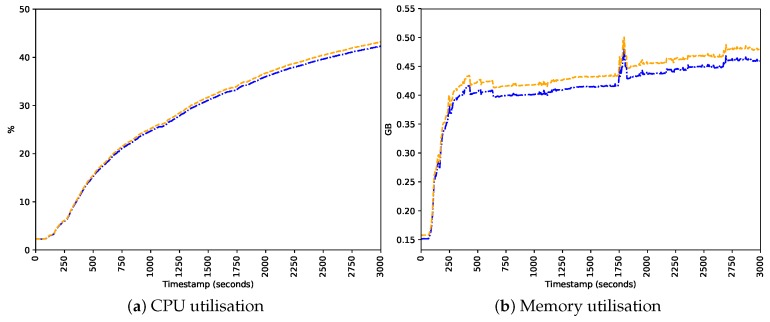

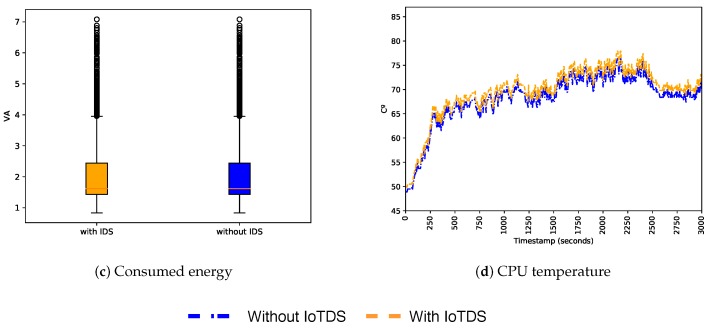
Behaviour of the MC profile infected with botnets regarding resource usage with and without the IoTDS.

**Figure 11:**
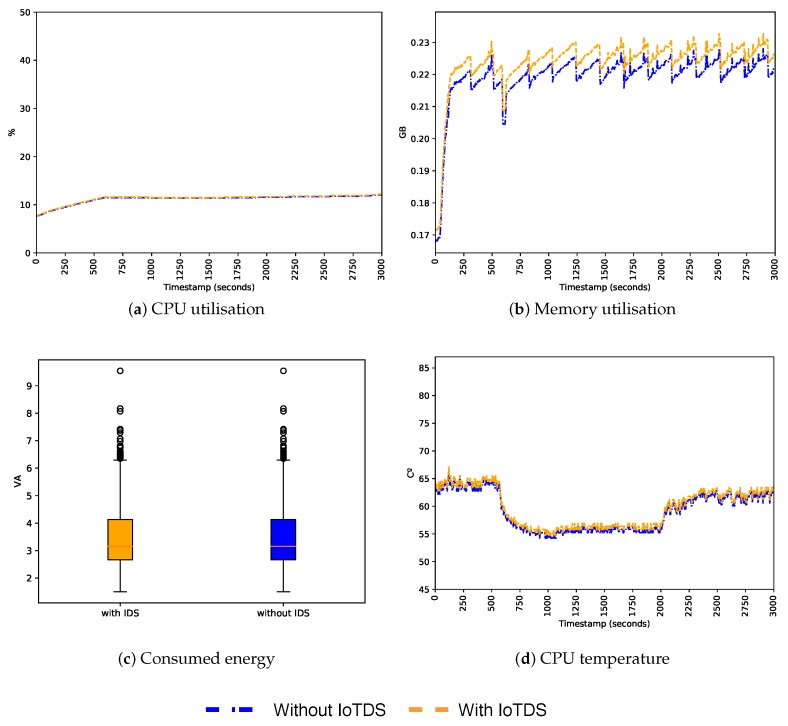
Behaviour of the SC profile infected with botnets regarding resource usage with and without the IoTDS.

**Figure 12:**
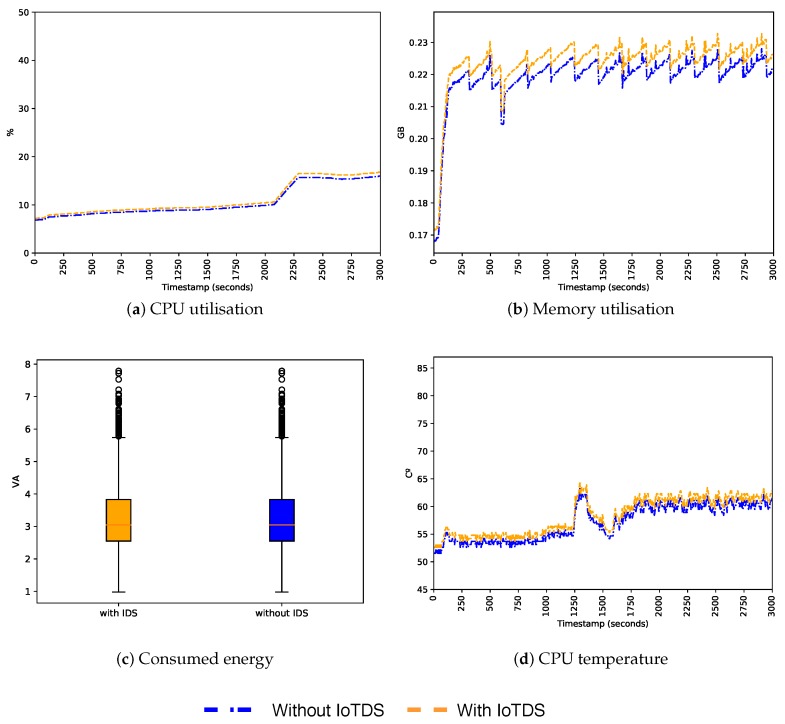
Behaviour of the ST profile infected with botnets regarding resource usage with and without the IoTDS.

For comparison purposes, we also analysed the impact of the IoTDS in a device infected by botnets. Figure 10, Figure 11 and Figure 12 present the CPU and memory utilisation, the energy consumption and the CPU temperature for all the profiles infected by the infection type 3. As shown in the figures, the behaviour was very similar in the cases with and without the IoTDS, as the botnets forced a high consumption of resources on the device. The MC profile showed the highest CPU and memory utilisation, compared to the SC and ST profiles. All resources presented intense use, regardless of the device was running the IoTDS.

In the SC profile, the memory and CPU utilisation were less intense, representing half of the memory and CPU used by the MC profile. The ST profile shows the same memory utilisation as the SC profile, but a higher CPU utilisation in some moments. In all tests, it is possible to see that the IoTDS impact in the device was almost the same in the infected and non-infected scenarios. This results showed that IoTDS can still be functional even with the device’s resources being heavily used by the botnets.

### 5.5. Discussion

Overall, all algorithms presented high predictive performance, detecting the seven botnets in all profiles. The different legitimate actions the device performed in the proposed profiles seemed to be distinguishable from malicious behaviours by most of the algorithms. All these good results were reached using only 1080 data instances to train the models and a 1-s time-window. It means that the system does not require long periods of training and can perform a persistent analysis. OSVM and LOF had the best performance out of the four tested classifiers, with LOF holding a slight advantage in most of the datasets.

The used host-based features showed to be accurate indicators of malware presence in the device. It is noteworthy to say that the adoption of host-based data does not rule out the possibility of using other types of data. Using host-based data in conjunction with network-based data, for example, can provide even more security for the entire system, including hosts and network. The impact of the IoTDS in the performance of the device was also considered. Our tests showed that although there was some increase in resource consumption when the IoTDS was running, this was far from hindering the capabilities of the device, regardless of its profile.

This work also has two limitations that are outlined as follows. Firstly, our approach does not handle concept drifts automatically. The normal behaviour of IoT devices can change as time passes, which may have multiple reasons, such as firmware updates or changes in user behaviour. To cope with this kind of situation, the proposed approach has to induce new models to the IoT devices, which cannot be done automatically for the time being. Secondly, the possibility of attacks against machine learning algorithms was not considered. This way, adversaries can try to poison the ML algorithms to make them classify malicious behaviours as legitimate.

Lastly, considering the memory and CPU utilisation of the Raspberry Pi during the tests, it is possible to say that the IoTDS could be a solution for multiple IoT devices found in today’s households. Examples of these IoT devices are: Odroid-XU4 (https://www.hardkernel.com/), which has a 2GB RAM and a Octa-Core processor, Roku TV (https://www.roku.com/en-gb/), which has a 512MB-1GB RAM in the 4k version with a ARM Quad-Core processor, and smart TVs such as the TCL L40S4900FS, which has a Quad-Core processor and a 1GB RAM.

## 6. Conclusions

This work proposes an approach named IoTDS to detect botnets in IoT devices. The IoTDS is a host-based solution, which analyses the host’s CPU and memory utilization, CPU temperature, and number of running tasks to classify its behaviour as malicious or legitimate. A one-class classifier performs the classification. The IoTDS architecture is organized into two elements: the IoTDS Agent, installed in the IoT device, and the IoTDS Management Console, installed in a separated server. In this proposed architecture, the effort of inducing new behaviour models is transferred to the Management Console, leaving the IoT devices free from running this costly task. In addition to data collection, the only responsibility of IoTDS Agents is to analyse new data using the behaviour model induced by the Management Console.

To evaluate the proposed approach, we used an experimental setup built in previous work. This setup includes a Raspberry Pi, which emulated three different IoT device profiles and was infected by seven different botnets. We organised the tests to evaluate the system’s predictive performance and its impact on the host. Four different one-class classifiers were used in our tests: EE, IF, LOF, and OSVM. Our tests showed that a sample size of 1,080 data instances for training and a 1-s time-window would be able to produce results as good as those obtained with larger training samples and time windows. After selecting the time window and the training sample size, we optimised the hyperparameters of all tested algorithms. The best algorithms were OSVM and LOF, with the LOF algorithm having a slight advantage with a mean F1-score of 94%. We also evaluated the impact of running the IoTDS in the three different IoT device’s profiles. These tests showed that the approach increases the CPU, memory, and energy utilisation of the device, but we did not detect any problems for the device’s operation.

As future work, we intend to evaluate the proposed approach in different devices and IoT botnets, including other host-based features (e.g., syscalls) and testing other ways of mitigating botnets, such as blocking IP addresses and changing IPs using software-defined networking routers in conjunction with the IoTDS. Also, we plan to make the system able to handle concept drifts and defend the machine learning algorithms from attacks against their detection capacity. Finally, employing federated machine learning techniques can be a promising alternative to make the system more resilient.

## Figures and Tables

**Table 1:** List of infections made in the Raspberry Pi profiles.

Infection Type	Profile	Botnet	Number of Samples
1	MC	Hajime	4
	SC	Aidra	11
	ST	BashLite	
2	MC	Mirai	1
	SC		
	ST		
3	MC	Mirai, Doflo, Tsunami, Wroba	28
	SC		
	ST		

**Table 2:** Features and their value ranges.

Feature	CPU Utilisation (%)	Memory Utilisation (GB)	Number of Tasks Running	CPU Temperature (°C)
Values	0–100	0–1	0–300	0–100

**Table 3:** Evaluation of time-window size for EE, IF, LOF and OSVM in all datasets.

Classifier	Time-Window Size (s)	Precision	Recall	Specificity	Accuracy	AUC	F1-Score
EE	1	0.6599	0.8187	0.9001	0.8837	0.8594	0.7267
	5	0.6879	0.8931	0.8992	0.8979	0.8961	0.7759
	10	0.6101	0.6883	0.8990	0.8566	0.7937	0.6394
	30	0.5542	0.6790	0.8984	0.8543	0.7887	0.5991
IF	1	0.5564	0.9848	0.7994	0.8366	0.8921	0.7100
	5	0.5746	0.9879	0.8147	0.8495	0.9013	0.7261
	10	0.5915	0.9746	0.8261	0.8560	0.9004	0.7342
	30	0.5434	0.9913	0.7877	0.8287	0.8895	0.7010
LOF	1	0.7266	0.9912	0.9062	0.9233	0.9487	0.8385
	5	0.7270	0.9893	0.9065	0.9231	0.9479	0.8380
	10	0.7248	0.9889	0.9055	0.9222	0.9472	0.8364
	30	0.7307	0.9939	0.9074	0.9248	0.9506	0.8419
OSVM	1	0.6903	0.8929	0.8999	0.8985	0.8964	0.7778
	5	0.6876	0.9183	0.8950	0.8997	0.9067	0.7854
	10	0.6848	0.8838	0.8993	0.8962	0.8916	0.7696
	30	0.6727	0.8797	0.8974	0.8938	0.8885	0.7578

**Table 4:** Evaluation of sample size for EE, IF, LOF and OSVM in all datasets.

Classifier	Sample Size	Precision	Recall	Specificity	Accuracy	AUC	F1-Score
EE	0.1	0.6617	0.8245	0.8998	0.8846	0.8621	0.7303
	0.2	0.6605	0.8206	0.9000	0.8840	0.8603	0.7279
	0.3	0.6598	0.8193	0.9001	0.8838	0.8597	0.7268
	0.4	0.6599	0.8195	0.8999	0.8837	0.8597	0.7269
	0.5	0.6601	0.8191	0.9001	0.8838	0.8596	0.7268
	0.6	0.6592	0.8175	0.9002	0.8835	0.8588	0.7256
	0.7	0.6593	0.8176	0.9002	0.8835	0.8589	0.7257
	0.8	0.6592	0.8182	0.9001	0.8836	0.8591	0.7259
	0.9	0.6591	0.8175	0.9001	0.8834	0.8588	0.7256
IF	0.1	0.5572	0.9856	0.7999	0.8372	0.8928	0.7109
	0.2	0.5583	0.9855	0.8009	0.8380	0.8932	0.7118
	0.3	0.5569	0.9856	0.7997	0.8370	0.8927	0.7107
	0.4	0.5594	0.9855	0.8014	0.8384	0.8934	0.7126
	0.5	0.5590	0.9855	0.8012	0.8382	0.8933	0.7123
	0.6	0.5582	0.9856	0.8007	0.8378	0.8931	0.7117
	0.7	0.5584	0.9853	0.8008	0.8378	0.8931	0.7118
	0.8	0.5581	0.9851	0.8007	0.8377	0.8929	0.7115
	0.9	0.5586	0.9855	0.8011	0.8382	0.8933	0.7121
LOF	0.1	0.7119	0.9835	0.8998	0.9166	0.9416	0.8257
	0.2	0.7169	0.9915	0.9015	0.9196	0.9465	0.8320
	0.3	0.7203	0.9919	0.9031	0.9210	0.9475	0.8345
	0.4	0.7227	0.9918	0.9043	0.9219	0.9481	0.8361
	0.5	0.7249	0.9915	0.9054	0.9227	0.9484	0.8375
	0.6	0.7271	0.9911	0.9065	0.9235	0.9488	0.8388
	0.7	0.7289	0.9904	0.9074	0.9241	0.9489	0.8397
	0.8	0.7313	0.9889	0.9086	0.9248	0.9488	0.8407
	0.9	0.7337	0.9873	0.9099	0.9255	0.9486	0.8418
OSVM	0.1	0.6882	0.8946	0.8986	0.8978	0.8966	0.7770
	0.2	0.6890	0.8927	0.8992	0.8979	0.8960	0.7769
	0.3	0.6896	0.8928	0.8995	0.8982	0.8962	0.7773
	0.4	0.6901	0.8932	0.8997	0.8984	0.8964	0.7778
	0.5	0.6903	0.8927	0.8999	0.8985	0.8963	0.7778
	0.6	0.6908	0.8931	0.9001	0.8987	0.8966	0.7782
	0.7	0.6904	0.8926	0.9000	0.8985	0.8963	0.7778
	0.8	0.6904	0.8929	0.9000	0.8985	0.8964	0.7779
	0.9	0.6904	0.8928	0.8999	0.8985	0.8964	0.7779

**Table 5:** Hyperparameters’ ranges evaluated to find the best performance.

Classifier	Hyperparameter	Range	Number of Samples
EE	contamination	[0, 0.5]	40
IF	n_estimators	(10, 50, 100)	3
	contamination	[0, 1)	100
LOF	n_neighbours	[1, 20]	20
	contamination	[0, 0.5)	20
OSVM (RBF, Linear)	γ	[0, 1)	20
	ν	[0, 1)	20
OSVM (Poly)	γ	[0, 1)	20
	ν	[0, 1)	20
	degree	(2, 3, 4, 5)	4

**Table 6:** Optimised hyperparameters.

Classifier	Hyperparameter	Value
EE	contamination	0.0650
IF	n_estimators	100
	contamination	0.0346
LOF	n_neighbours	12
	contamination	0.0091
OSVM	γ	0.8941
	ν	0.0166
	kernel	‘rbf’

## Abbreviations

The following abbreviations are used in this manuscript:

C&C	Command and Control
CoAP	Constrained Application Protocol
CPU	Central Processing Unit
DDoS	Distributed Denial of Service
DHCP	Dynamic Host Configuration Protocol
DNS	Domain Name System
EE	Eliptic Envelope
FAST-MCD	Fast Minimum Covariance Determinant
HTTP	Hypertext Transfer Protocol
HTTPS	Hypertext Transfer Protocol Secure
IDS	Intrusion Detection System
IF	Isolation Forest
IoT	Internet of Things
LDA	Linear Discriminant Analysis
LRD	Local Reachability Distance
MC	Multimedia Centre Profile
ML	Machine Learning
LOF	Local Outlier Factor
OSVM	One-class Support Vector Machine
PCA	Principal Component Analysis
RBF	Radial Basis Function
RAM	Random Access Memory
RD	Reachability Distance
RPL	Routing Protocol for Low Power and Lossy Networks
SC	Surveillance Camera Profile
SSH	Secure Shell
ST	Surveillance Camera with Additional Traffic Profile
SVM	Support Vector Machine
6LoWPAN	IPv6 over Low-Power Wireless Personal Area Networks

## References

[1] Ashton K. That ‘Internet of Things’ Thing. https://www.rfidjournal.com/articles/view?4986.

[2] Ghayvat H., Mukhopadhyay S., Gui X., Suryadevara N. (2015). WSN- and IOT-Based Smart Homes and Their Extension to Smart Buildings. Sensors.

[3] Shi X., An X., Zhao Q., Liu H., Xia L., Sun X., Guo Y. (2019). State-of-the-Art Internet of Things in Protected Agriculture. Sensors.

[4] Navarro-Ortiz J., Sendra S., Ameigeiras P., Lopez-Soler J.M. (2018). Integration of LoRaWAN and 4G/5G for the Industrial Internet of Things. IEEE Commun. Mag..

[5] Schleicher J.M., Vögler M., Dustdar S., Inzinger C. (2016). Application Architecture for the Internet of Cities: Blueprints for Future Smart City Applications. IEEE Internet Comput..

[6] Portilla J., Mujica G., Lee J., Riesgo T. (2019). The Extreme Edge at the Bottom of the Internet of Things: A Review. IEEE Sens. J..

[7] Ibarra-Esquer J.E., González-Navarro F.F., Flores-Rios B.L., Burtseva L., Astorga-Vargas M.A. (2017). Tracking the Evolution of the Internet of Things Concept Across Different Application Domains. Sensors.

[8] Kolias C., Kambourakis G., Stavrou A., Voas J. (2017). DDoS in the IoT: Mirai and Other Botnets. Computer.

[9] Yu S., Wang G., Liu X., Niu J. (2018). Security and Privacy in the Age of the Smart Internet of Things: An Overview from a Networking Perspective. IEEE Commun. Mag..

[10] Angrishi K. (2017). Turning Internet of Things(IoT) into Internet of Vulnerabilities (IoV): IoT Botnets. arXiv.

[11] Bertino E., Islam N. (2017). Botnets and Internet of Things Security. Computer.

[12] Zarpelão B.B., Miani R.S., Kawakani C.T., de Alvarenga S.C. (2017). A survey of intrusion detection in Internet of Things. J. Netw. Comput. Appl..

[13] Raza S., Wallgren L., Voigt T. (2013). SVELTE: Real-time intrusion detection in the Internet of Things. Ad Hoc Netw..

[14] Amaral J.P., Oliveira L.M., Rodrigues J.J., Han G., Shu L. Policy and network-based intrusion detection system for IPv6-enabled wireless sensor networks. Proceedings of the 2014 IEEE International Conference on Communications (ICC).

[15] Granjal J., Silva J.M., Lourenço N. (2018). Intrusion Detection and Prevention in CoAP Wireless Sensor Networks Using Anomaly Detection. Sensors.

[16] Le A., Loo J., Chai K.K., Aiash M. (2016). A Specification-Based IDS for Detecting Attacks on RPL-Based Network Topology. Information.

[17] Alrashdi I., Alqazzaz A., Aloufi E., Alharthi R., Zohdy M., Ming H. AD-IoT: Anomaly Detection of IoT Cyberattacks in Smart City Using Machine Learning. Proceedings of the 2019 IEEE 9th Annual Computing and Communication Workshop and Conference (CCWC).

[18] Jan S.U., Ahmed S., Shakhov V., Koo I. (2019). Toward a Lightweight Intrusion Detection System for the Internet of Things. IEEE Access.

[19] Habibi J., Midi D., Mudgerikar A., Bertino E. (2017). Heimdall: Mitigating the Internet of Insecure Things. IEEE Internet Things J..

[20] Meidan Y., Bohadana M., Mathov Y., Mirsky Y., Breitenbacher D., Shabtai A., Elovici Y. (2018). N-BaIoT: Network-based Detection of IoT Botnet Attacks Using Deep Autoencoders. arXiv.

[21] Bezerra V.H., da Costa V.G.T., Martins R.A., Barbon Junior S., Miani R.S., Zarpelão B.B. (2018). Providing IoT host-based datasets for intrusion detection research. SIMPÓSIO BRASILEIRO EM SEGURANÇA DA INFORMAÇÃO E DE SISTEMAS COMPUTACIONAIS (SBSEG), 2018 Anais do XVIII Simpósio Brasileiro em Segurança da Informação e de Sistemas Computacionais.

[22] Bezerra V.H., da Costa V.G.T., Barbon Junior S., Miani R.S., Zarpelão B.B. (2018). One-class Classification to Detect Botnets in IoT devices. SIMPÓSIO BRASILEIRO EM SEGURANÇA DA INFORMAÇÃO E DE SISTEMAS COMPUTACIONAIS (SBSEG), 2018 Anais do XVIII Simpósio Brasileiro em Segurança da Informação e de Sistemas Computacionais.

[23] An N., Duff A., Naik G., Faloutsos M., Weber S., Mancoridis S. Behavioral anomaly detection of malware on home routers. Proceedings of the 2017 12th International Conference on Malicious and Unwanted Software (MALWARE).

[24] Khan S.S., Madden M.G. (2009). A survey of recent trends in one class classification. Artificial Intelligence and Cognitive Science.

[25] Rousseeuw P.J., Driessen K.V. (1999). A fast algorithm for the minimum covariance determinant estimator. Technometrics.

[26] Liu F.T., Ting K.M., Zhou Z.H. Isolation forest. Proceedings of the 2008 Eighth IEEE International Conference on Data Mining.

[27] Breunig M.M., Kriegel H.P., Ng R.T., Sander J. LOF: identifying density-based local outliers. Proceedings of the 2000 ACM SIGMOD International Conference on Management of Data.

[28] Cortes C., Vapnik V. (1995). Support-vector networks. Mach. Learn..

[29] Resende P.A.A., Drummond A.C. (2018). A Survey of Random Forest Based Methods for Intrusion Detection Systems. ACM Comput. Surv..

[30] Shin H.J., Eom D.H., Kim S.S. (2005). One-class support vector machines—An application in machine fault detection and classification. Comput. Ind. Eng..

[31] Hoyle B., Rau M.M., Paech K., Bonnett C., Seitz S., Weller J. (2015). Anomaly detection for machine learning redshifts applied to SDSS galaxies. Mon. Not. R. Astron. Soc..

[32] Stallings W. (2017). Cryptography and Network Security: Principles and Practice.

[33] Antonakakis M., April T., Bailey M., Bernhard M., Bursztein E., Cochran J., Durumeric Z., Halderman J.A., Invernizzi L., Kallitsis M. Understanding the Mirai Botnet. Proceedings of the 26th USENIX Security Symposium.

[34] Stavrou A., Voas J., Fellow I. (2017). DDoS in the IoT. Computer.

[35] Abdul Kadir A.F., Stakhanova N., Ghorbani A.A., Qiu M., Xu S., Yung M., Zhang H. (2015). Android Botnets: What URLs are Telling Us. Network and System Security.

[36] Pedregosa F., Varoquaux G., Gramfort A., Michel V., Thirion B., Grisel O., Blondel M., Prettenhofer P., Weiss R., Dubourg V. (2011). Scikit-learn: Machine Learning in Python. J. Mach. Learn. Res..

[37] Sokolova M., Lapalme G. (2009). A systematic analysis of performance measures for classification tasks. Inf. Process. Manag..

[38] Bergstra J., Bengio Y. (2012). Random search for hyper-parameter optimization. J. Mach. Learn. Res..

